# Isotopically Selected Co‐Doping of ^121^Sb and ^123^Sb Pairs in Silicon

**DOI:** 10.1002/adma.202522240

**Published:** 2026-03-02

**Authors:** Mason Adshead, Maddison Coke, Evan Tillotson, Tomas F Bouvier, Artem Mkrtychyan, Kexue Li, Sam Sullivan‐Allsop, Ricardo Egoavil, William Thornley, Yi Cui, Christopher M. Gourlay, Katie L Moore, Flyura Djurabekova, Sarah J Haigh, Richard J Curry

**Affiliations:** ^1^ Department of Electrical and Electronic Engineering Photon Science Institute University of Manchester Manchester UK; ^2^ Department of Materials Photon Science Institute University of Manchester Manchester UK; ^3^ Department of Physics University of Helsinki Helsinki Finland; ^4^ Thermo Fisher Scientific Eindhoven The Netherlands; ^5^ Department of Materials Imperial College London London UK

**Keywords:** quantum computing, qubit, qudit

## Abstract

A reliable route to the deterministic fabrication of impurity ion donors in silicon is required to advance quantum computing architectures based upon such systems. This paper reports the ability to dope isotopically‐defined unique (^121^Sb^123^Sb)^2+^ molecular ions into silicon with measured detection efficiencies of 94% being obtained. Atomically resolved imaging of the doped Sb ions reveals substitutionally incorporated atoms with a Sb‐to‐Sb separation of ≈2 nm post‐implantation, thus indicating suitability to form coupled qudit systems. Molecular dynamics simulations support the preference for doped Sb atoms to occupy lattice sites, driven by fast (≈1s) re‐crystallization of localized ion implantation induced damage at 300 K. The Sb doping method used is fully compatible with integration into processing that includes pre‐enrichment of the silicon host to sub‐3 ppm ^29^Si levels. As such, we present a potential pathway to the creation of scaled qudit arrays within silicon platforms for quantum computing.

## Graphical abstract

Pairs of ^121^Sb and ^123^Sb atoms are doped into silicon using single cluster implantation and found to reside substitutionally within the crystal lattice due to self‐annealing with a Sb‐to Sb separation of ≈2 nm. Molecular dynamics simulations reproduce the observed experimental results and single ion cluster detection efficiencies of 94% are demonstrated.

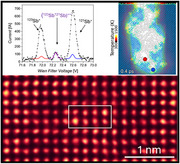

## 1 Introduction

The development of a quantum computer that fully realizes the potential of quantum‐based operations requires a scalable material platform to facilitate its fabrication. This presents a significant challenge as an error‐corrected machine will require 10^4^ to 10^6^ physical qubits, depending on their performance and operation. A variety of approaches are being explored to deliver this based upon various technology platforms, including trapped ion [1, 2, 3] superconducting [4, 5, 6], optical [7] and semiconductor [8, 9, 10, 11] based qubit systems. Each approach has its own advantages and challenges [12, 13], but in terms of scaling to large qubit numbers, semiconductor platforms are attractive due to opportunities to leverage the mature state of microelectronics processing.

Significant effort has therefore been expended in developing Si‐based architectures for quantum computing with electrostatically gate‐defined quantum dots and impurity ion donors, both yielding qubit devices [14]. The former, utilizing a large (232) ensemble of 12 Si:SiGe quantum dot arrays, have demonstrated average coherence times (*T*_2_^*^) of 0.6 µs in natural Si (^nat^Si). This was increased by an order of magnitude through reducing the residual ^29^Si isotopic population from that of ^nat^Si (≈46,000 ppm) to ≈800 ppm, which reduces nuclear spin‐related decoherence. The use of impurity ion donors, proposed by Kane [15], has delivered electron coherence values of *T*_2_^*^ ≈55 ns in ^31^P‐doped ^nat^Si, again limited by the residual ^29^Si nuclear spin interactions [16]. Harnessing of the nuclear spin (*I*) of such donors has also been demonstrated with values of T_2_^*^ ≈ 0.84 ms and 3 ms for the neutral and charged donors (D^0^ and D^+^) respectively, where *I* = 1/2 for ^31^P [17]. These coherence times are also limited by the presence of ^29^Si hence efforts to produce increasingly isotopically pure ^28^Si have been pursued, with record enrichment levels of ≈2 ppm ^29^Si being recently reported through focused ion beam enrichment [18]. Recently the demonstration of an 11‐qubit atom processor based upon ^31^P doped enriched Si has realized two‐qubit gate fidelities in silicon of 99.9% [19].

The utilization of donor ions enables the combined use of both the electron and nuclear spin to access qubits operating in a higher dimensional Hilbert space. This scales with *I* of the donor which for ^31^P (*I* = 1/2) provides a 4D system when coupled to the electron spin (*S* = 1/2). The utilization of higher nuclear spin donors such as ^123^Sb (*I* = 7/2) has been demonstrated to enable access to qudits operating in 16D space [20]. A single such qudit has recently been used to demonstrate ‘Schrodinger's cat’ entangled superposition states [21]. The utilization of qudits therefore provides a powerful opportunity for inward scaling of qubits which can then be combined with physical scaling of such systems. The gain of using a *d*‐dimensional qudit over a 2D qubit is a reduction by log_2_ *d* in the number of physical units required, a factor of 4 for ^123^Sb. Furthermore, coupling together two such donor systems yields a (log_2_ *d*)^2^ improvement. Qudits are also of interest due to additional functionality they may provide such as self‐cooling that has been reported in molecular systems [22].

The potential for such scaling presents challenges and opportunities. If successfully implemented it could significantly reduce the architecture footprint of future quantum devices whilst accelerating performance. However, creating coupled qudits requires the ability to reproducibly manufacture them with high precision. Prior work using atomic‐force microscopy as a stenciling tool has demonstrated array formation [23]. However, the process used requires the pre‐fabrication of p‐i‐n junction formation within the substrate to detect implanted species. This adds cost, complexity and ultimately limits the scalability of the method to large chip areas where donor arrays of ≈1 million are required. Here we demonstrate the use of focused ion beam (FIB) implantation to dope Si with pairs of ^121^Sb and ^123^Sb donors, with a detection efficiency of 94% without the requirement for detection device fabrication. We use NanoSIMS characterization to confirm the isotopic nature of the implanted Sb atoms, allowing us to utilize a unique (^121^Sb^123^Sb)^2+^ molecular ion. We achieve reproducible Sb‐Sb lateral separation of sub‐3 nm and directly image these with atomic resolution over an extended field of view for the first time. Though we do not anneal the doped Si, we note that all Sb‐ions reside substitutionally on Si lattice sites and we do not observe ion implantation‐induced damage. This is understood through performing advanced molecular dynamics modelling which demonstrate room‐temperature annealing occurs on short (≈1 s) timescales.

## 2 Results and Discussions

**FIGURE 1:**
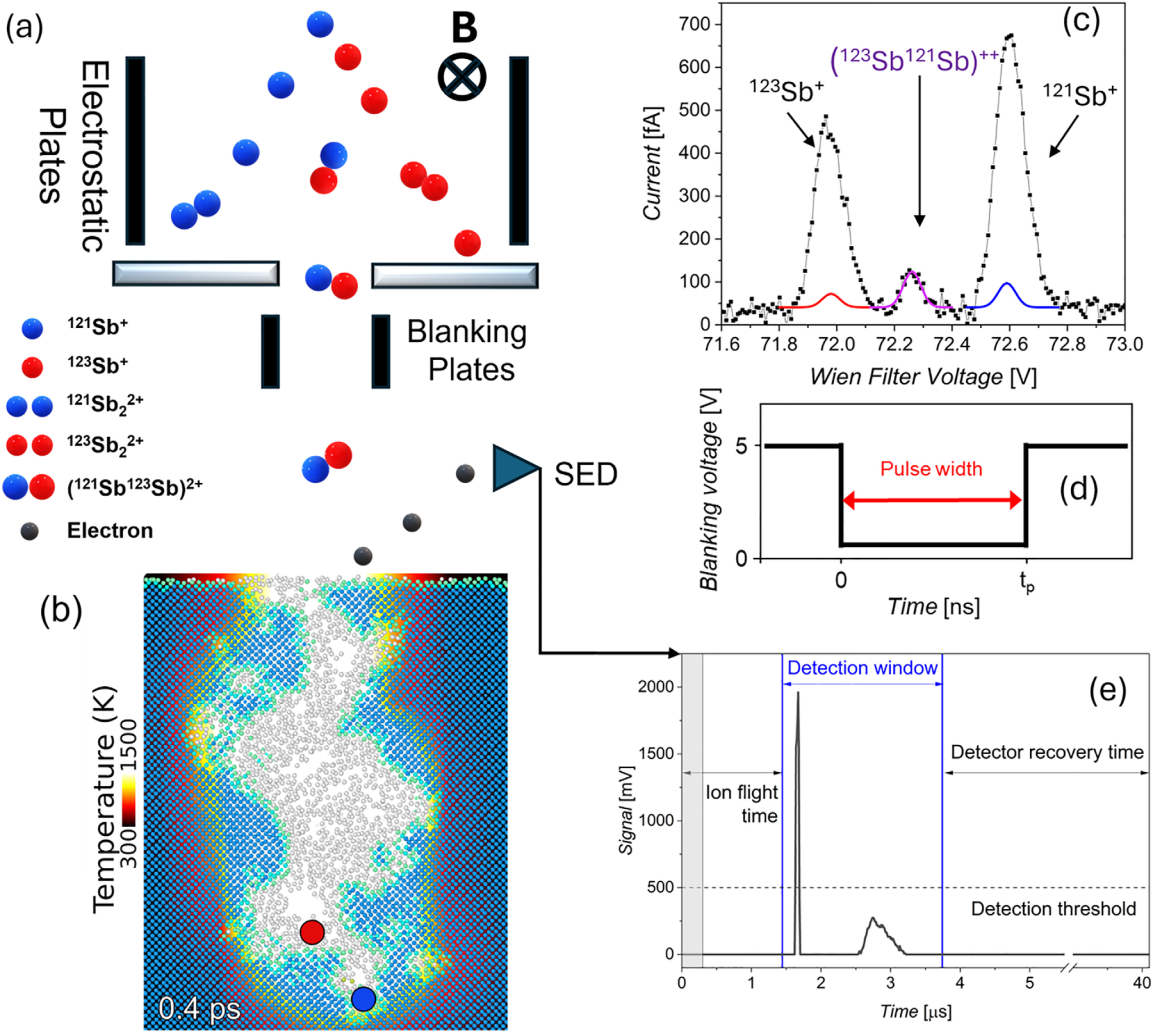
(a) Schematic of Wien filter (WF) mass‐selection of (^121^Sb^123^Sb)^2+^ ions and detection of emitted secondary electrons from implantation events. (b) Molecular dynamic simulation snapshot of damage cascade induced by a (^121^Sb^123^Sb)^+2^ ion at 30 keV in Si (also see Figure S1). (c) WF voltage scan of the ion current (using 15 kV acceleration) evidencing a central peak due to the emission of (^121^Sb^123^Sb)^+2^ ions. The measured data (black squares) are fitted based upon the isotopic native abundance (colored lines). (d) Schematic of ion‐beam pulsing use to obtain single‐ion operation. (e) Example secondary electron detection signal correlating with single ion implantation event.

Initial observation of the potential to isolate Sb_2_ molecules within a FIB was first noted by Adshead et al. [24]. To our knowledge, the isolation and verification of such a molecular ion has not previously been achieved, due to a combination of reasons, which include low mass resolution instrumentation [25]. Indeed, it was not until MC‐ICP‐MS (multicollector inductively coupled plasma mass spectrometry) was available that the relative abundance of the two naturally occurring Sb isotopes (^121^Sb and ^123^Sb) could be verified [26]. Here, the Platform for Nanoscale Advanced Materials Engineering (P‐NAME) FIB Facility (Q‐One, Ionoptika) is used at a 15 kV anode voltage with a liquid metal Au_x_Si_y_Sb_z_ eutectic alloy ion source. A schematic of the ion species mass separation through control of a Wien Filter (WF) voltage is shown in Figure 1a along with the detection of secondary electrons resulting from ion implantation of a (^121^Sb^123^Sb)^2+^ molecular ion into Si. Figure 1b shows a snapshot (0.4 ps after impact) of the damage cascade induced by a (^121^Sb^123^Sb)^+2^ ion at 30 keV in Si. The positions of the two now separated ions is indicated and does not change further with time (Figure S1).

It is important to note that whilst we use the molecular ion to select and implant the ^121^Sb and ^123^Sb pair, it does not remain a molecule upon implantation. This is immediately apparent upon comparing the ≈300 kJ mol^−1^ (≈3.1 eV) binding energy of the Sb_2_ molecule [27] with the implantation energy of 30 keV that is 4 orders of magnitude larger. As such, from the point of entering the Si each Sb ion can be considered independent and treated as if two single ions had been independently implanted at the same point with an energy of 15 keV each. In practice the alternative approach of sequentially implanting two ions at the same point on the silicon surface is however not feasible given the spatial resolution of the ion beam (defining the probability of a single ion being ‘found’ at a given point) which for the energies used in this study is 60 nm. The use of the molecular ion therefore has the additional advantage of overcoming the inherent challenge of implanting two ions at the same position of a surface.

A Wien filter (WF) spectrum showing the fully resolved Sb peaks is shown in Figure 1c with the full WF spectrum shown in Figure S2). The high mass (*M*) resolving power of at this mass‐energy range (*R* = *M*/Δ*M* = 296 ± 7) enables the identification of an additional peak lying between the ^121^Sb and ^123^Sb peaks. This additional third peak exhibits a significantly lower ion current, and has no potential isobars which may be identified to account for its presence. It is therefore proposed (and later shown) that the peak is due to the presence of a (^121^Sb^123^Sb)^2+^ molecular ion. This implies that associated monoisotopic ^121^Sb_2_^2+^ and ^123^Sb_2_^2+^ molecular ions are present within the equivalent isotopic singly‐charged ion (Sb^+^) peaks, which cannot be separated. Using the natural abundance of the Sb isotopes [28], integration of the peaks in Figure 1c yields ratios for the proposed molecular ions of 18.2:48.4:32.7, respectively, for ^123^Sb_2_^2+^: (^121^Sb^123^Sb)^2+^:^121^Sb_2_^2+^, these are modelled in Figure 1c as colored solid lines.

The advantage of utilizing the (^121^Sb^123^Sb)^2+^ molecular ion in this work is the certainty that each implantation event delivers precisely two Sb atoms. As ^122^Sb is a synthetic radioactive isotope with a ≈2.7 d half‐life there is no possible mass to charge ratio isobar of the (^121^Sb^123^Sb)^2+^ molecular ion (e.g., ^122^Sb^+^) therefore ruling out unintended single Sb atom implantation. In contrast the presence of ^121^Sb_2_^2+^ and ^123^Sb_2_^2+^ molecular ions has implications for attempting single Sb ion implantation using singly‐charged isotopes (^121^Sb^+^ and ^123^Sb^+^) as these are indistinguishable from the doubly‐charged molecular ion Sb pairs of the same isotope, which would be present with a 6–8% occurrence. Therefore, for applications requiring single‐ion Sb qudit doping isotopically selected Sb^2+^ ions should be used where there is no evidence of an associated isobar molecular ion with the same mass to charge ratio (e.g. ^123^Sb_2_^4+^) as shown in Figure S2.

There is a further potential advantage of utilizing pairs of Sb ions consisting of the different isotopes for future qubit devices. The differing nuclear spins of the ^121^Sb and ^123^Sb isotopes (*I* = 5/2 and *I* = 7/2 respectively) result in different hyperfine coupling occurring to the electronic spin. This results in differing electron spin resonance frequencies, *f*, for the transitions associated with each isotope, which provides additional versatility in terms of control. For example, the clock transitions (of interest due to the reduced sensitivity of transition energy to external magnetic field, *B*, variation as to first order d*f*/d*B* = 0) for the ^121^Sb transitions Δ*m_S_* = +1, Δ*m_I_* = −1/2 and −3/2 occur at *f* = 3.21 GHz and *f* = 0.92 GHz respectively. Whilst for the ^123^Sb transitions Δ*m_S_* = +1, Δ*m_I_* = −1/2, −3/2 and −5/2 they occur at *f* = 3.17 GHz, *f* = 0.98 GHz and *f* = 0.49 GHz [29]. This therefore overcomes the degeneracy that would be associated with using pairs of identical isotopes, allowing each Sb donor to be independently addressed.

**FIGURE 2:**
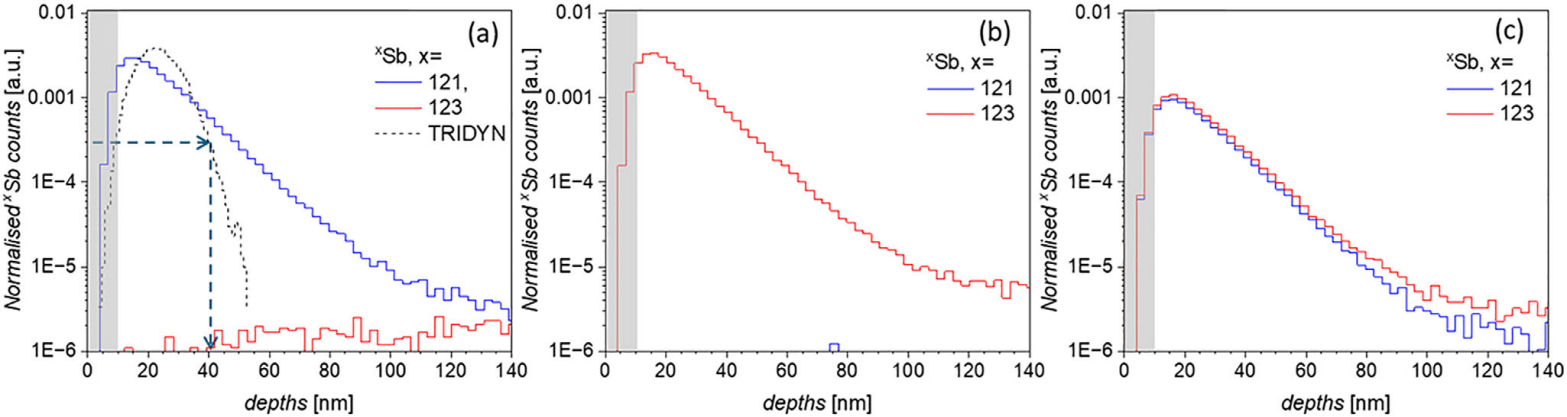
NanoSIMS depth profiles of ^121^Sb and ^123^Sb, normalized to the bulk native ^28^Si count obtained from the sample. (a) ^121^Sb^+^ (b) ^123^Sb^+^ and (c) (^121^Sb^123^Sb)^2+^ molecular ion implantation at 25 kV anode. (a) also shows the simulated TRIDYN profile for Sb^+^ 25 keV 1 × 10^15^ ions cm^−2^ implantation (blue dashed line). The depths were calibrated using post‐NanoSIMS atomic force microscopy. The grey shaded region at shallow depths indicates the pre‐steady state NanoSIMS collection regime, and the dashed arrows indicate the lower concentration limit beyond which TRIDYN is not adapted to model.

Confidence in the identification of the two Sb peaks comes from their position in the Wien filter scan and analysis of their relative areas, with the two peaks being 57.2% and 42.8% consistent with published abundance values (57.21% and 42.79%) for ^121^Sb and ^123^Sb [30]. To confirm this a 5 µm × 5 µm region of an intrinsic Si wafer was implanted with singly charged (25 keV) Sb to a dose of 1 × 10^15^ ions cm^−2^ at each Wien filter voltage associated with the three peaks observed in Figure 1c. Figure 2a,b shows NanoSIMS depth profiles for the ^121^Sb^+^ and ^123^Sb^+^ implanted regions, with only a single Sb isotope being found in each case. Figure 2c displays the NanoSIMS depth profiles for the (^121^Sb^123^Sb)^2+^ implantation using the same dose as in Figure 2a,b. This clearly records the presence of both Sb isotopes (each with a reduced relative concentration compared to the monoisotopic regions). All counts are normalized to the ^28^Si count level found in the ^nat^Si wafer at long range. It is worth noting the NanoSIMS obtained depth profile of the implantation does not vary significantly between singly‐charged single Sb ion and doubly‐charged Sb_2_ molecular ion implantation, indicating that the energy spread of the implanted ions, centered around 25 keV for each ion, is small as is to be expected.

The Sb doping profile of 1 × 10^15^ ions cm^−2^ at 25 keV into Si was simulated using TRIDYN, a dynamic Monte Carlo simulation for ion implantation [31], and is compared to the measured NanoSIMS depth profiles of ^121^Sb and ^123^Sb, in Figure 2a. It is noted that TRIDYN is not adapted to model concentrations below 0.1% (equating to a normalized count of ≈3 × 10^−4^ in Figure 2a) meaning beyond a depth of ≈40 nm the predicted concentration should be treated with caution. The simulation predicts a peak in the Sb doping concentration at a depth of ≈22 nm, which is consistent with the experimentally measured Sb peak implantation depth of ≈18 nm. This comparison takes into account the uncertainty associated with the ≈10 nm surface accumulation layer necessary for steady state NanoSIMS analysis previously discussed [18]. However, noting the limitation of TRIDYN, the simulations predict a faster decay of the implantation profile than is observed experimentally. It is possible that ‘knock‐on’ ion effects are extending the Sb concentration deeper into the sample. Such knock‐on implantation effect may result either from the NanoSIMS ion (Cs^+^) bombardment during analysis, or simply during Sb implantation as a result of the high‐dose rate used to achieve sufficient Sb concentrations for these tests. The latter will not affect use of Sb in qudit applications as Sb would be implanted as a single molecular ion and the former is a measurement artefact, suggesting the TRIDYN simulations can be taken as a guide for of the statistical depth distribution of implanted Sb molecular ions.

The NanoSIMS analysis in Figure 2c has demonstrated the controlled implantation of equal amounts of ^121^Sb and ^123^Sb using Sb_2_ molecular ions, but the ≈50 ppm detection limit of NanoSIMS is too low to allow measurement of a single Sb_2_ molecule required for qudit fabrication. Aberration corrected STEM imaging is one of the only techniques able to detect single Sb molecules buried within the implanted film. To avoid the potential artefacts that could be created by cross sectional sample preparation, (^121^Sb^123^Sb)^2+^ molecular ions were directly implanted into pre‐prepared 20 nm thick Si membranes (see Figure S3) at an energy of 30 keV (15 kV anode voltage). TRYDIN simulations indicate that at this voltage ≈76% of implanted Sb atoms will stop within the membrane. Single molecular ion doping of the membrane was undertaken using a pulsed ion beam with the current and pulse width set to achieve one Sb_2_ molecular ion per pulse. The FIB implantation ‘spot‐size’ was 60 nm (determined using the edge method previously described [24]) and single pulses were implanted with a 15 nm step size with the aim of implanting a Poissonian distribution of isolated pairs of Sb atoms suitable for STEM imaging.

**FIGURE 3:**
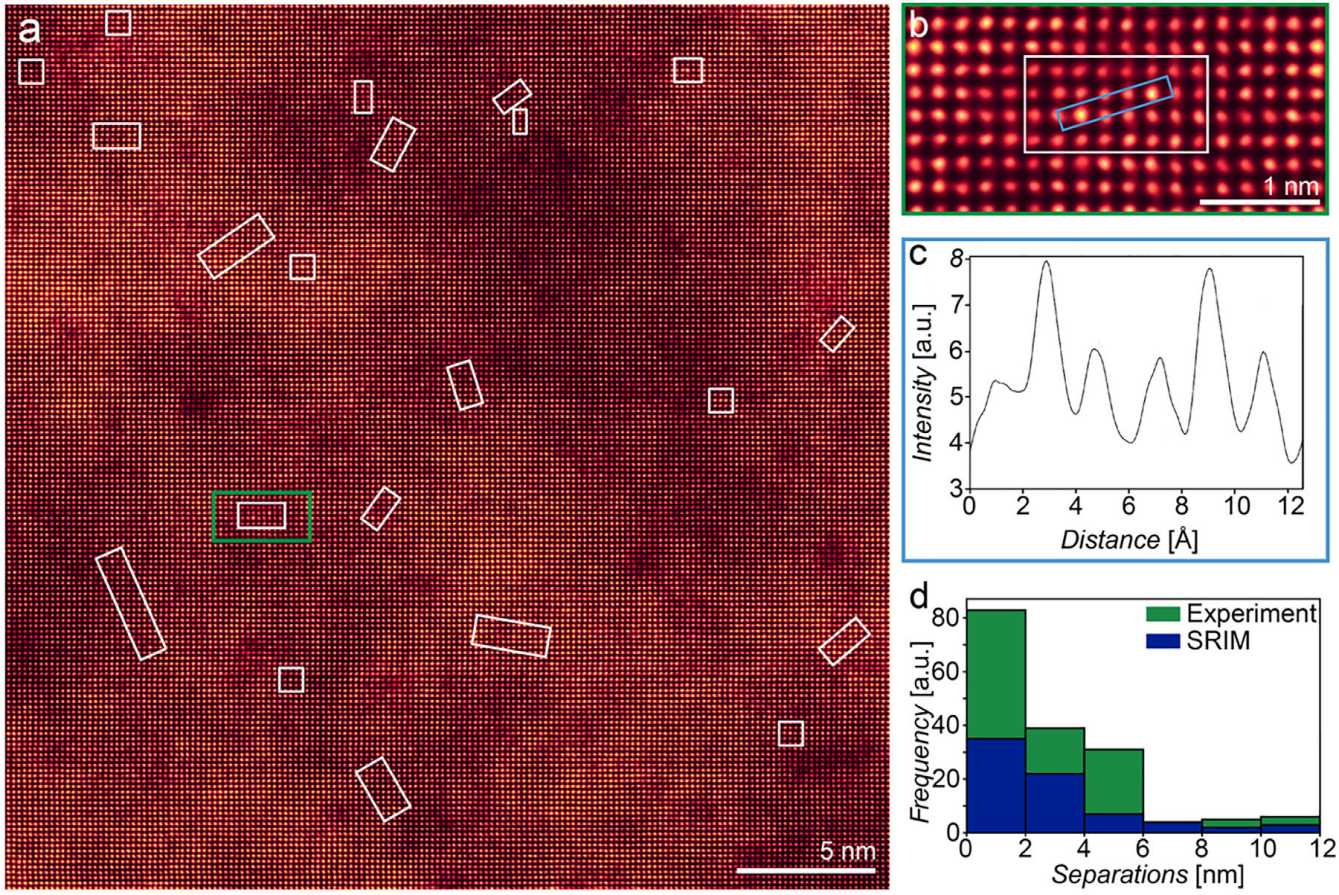
ADF STEM imaging of Sb‐implantation in Si. (a, b) ADF STEM images of Si membrane highlighting the presence of Sb ions resulting from implantation using single (^121^Sb^123^Sb)^2+^ molecular ions with a planar spacing of 6.2 Å. Single‐ and double‐ion dopants are highlighted via white squares and rectangles, respectively. (c) Intensity line profile demonstrating the ≈33% increase in peak intensity resulting from substitution of a single Sb ion into the Si lattice. (d) Histograms showing the experimentally measured Sb‐to‐Sb separation compared with the expected distribution predicted from SRIM Monte Carlo simulations [32].

Annular dark field (ADF) STEM imaging of the implanted membrane is shown in Figure 3a, where pairs of Sb atoms are highlighted via white rectangles. Implanted Sb atoms are visible due to the higher atomic number of Sb compared to the surrounding Si lattice, with a local ≈33% increase in the ADF intensity for a Sb substituted Si column measured experimentally compared to the surrounding lattice (Figure 3c). Larger magnitude, but longer period, intensity variations are also observed in the Si lattice, but these are due to thickness variations in the membrane as well as the presence of surface contamination. Multislice image simulations show that to optimize imaging conditions, a semi convergence angle of 21 mrad with ADF collection angles of 30–50 mrad (Figure S4) is needed. The predicted average ADF contrast of single Sb dopants in 20 nm thick Si is 34%, consistent with the experimental observations (see Figures S5 and S6). Imaging the nearest neighbor Sb‐to‐Sb distances for 176 dopant atoms revealed a peak Sb–Sb distance of ≈2 nm (Figure 3d) consistent with that expected for the implantation of (^121^Sb^123^Sb) molecular ions at this energy. Through increasing the implantation energy it would be possible to increase the average Sb‐to‐Sb distance, though this would be accompanied by an increase the spread of separation values and in the average depth. A further 30 isolated atomic dopants were identified where no other Sb peak was observed within the 1445 nm by 1445 nm field of view. For these isolated atoms the pair from the molecular ion is expected to be either one of the 24% that are predicted to have passed through the film during implantation, or to be at a sufficiently different depth in the sample that due to the ≈10 nm depth of field of the STEM image, they are not visible in a single frame.

Within the ADF STEM images of the Sb‐doped silicon membrane it is noticeable that (i) no evidence of ion induced damage is observed, and, (ii) all Sb atoms are found to be substitutionally incorporated within the Si crystal structure. We emphasize that no post implantation thermal annealing process has been performed prior to imaging, hence at first sight these findings are unexpected. Ion implantation of (^121^Sb^123^Sb)^2+^ molecular ions with an energy of 30 keV will inevitably lead to structural damage of the Si, and the defects formed during the impact may interact with dopants in a post‐collision stage, potentially passivating qubits. However, the localized amorphous pockets and defect clusters created are known to recrystallize during the post‐collision phase [33]. To verify the probability of defect survival following implantation we performed a series of molecular dynamic (MD) simulations of (^121^Sb^123^Sb)^2+^ molecular ion implantation into a Si crystal using the same energy as used in the experiment (30 keV). In our simulations the two Sb ions within the cluster produced collectively a total of 4505 ± 364 defects per cascade.

A series of snapshots of the evolution of the damage produced by a (^121^Sb^123^Sb)^+2^ molecular ion at 30 keV in Si, taken at 0.4, 5.4, 10 and 15 ps following implantation, is shown in Figure S1. White atoms represent defects identified via Diamond Structure analysis in OVITO (see Methods). A temperature heat map is superimposed on the particle slice. At 0.4 ps, the thermal spike elevates the local temperature well beyond the Si melting point, with some regions exceeding the boiling threshold. This extreme heating, combined with significant atomic displacements, leads to the formation of large voids. By 5.4 ps, the vaporized silicon has cooled to below the boiling point but remains above the melting threshold, forming a liquid inclusion within the Si lattice. At 10 ps, the liquid region cools to below the melting point but remains thermally elevated. Finally, by 15 ps, most regions have cooled to near ambient conditions, with residual hotspots found at ≈500 K. At this point the amorphous pocket has stabilized in shape and size, indicating the formation of stable damage on MD timescales. The continuing evolution of the system over these timescales is also observed by simply recording the number of defects as a function of time which shows that defect counts continue to evolve beyond 6 ps (Figure S1b). By 15 ps it is seen that fluctuations in the defect count reduce to a pseudo‐steady‐state on these short timescales, justifying the chosen simulation endpoint.

**FIGURE 4:**
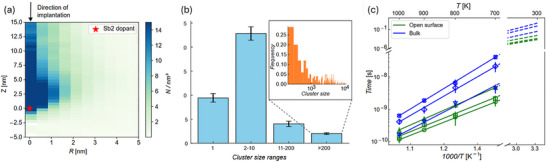
MD analysis of the damage evolution after a (^121^Sb^123^Sb)^2+^ molecular ion implantation impact into Si. (a) Spatial distribution of the number of defects with respect to the final position of a Sb dopant, immediately after the cascade. In this distribution, each of the two implanted Sb atoms is plotted as an individual dopant. (b) Defect cluster size distribution immediately after the collision cascade, the inset shows the frequency of large defect clusters of the given size to form during a single molecular ion impact. (c) Temperature dependence of the recrystallization time for selected defect clusters to shrink to one‐tenth of their original size. The clusters are randomly selected either close to the surface (green lines) and in the bulk (blue lines). Temperature dependence of each cluster was fitted with an individual Arrhenius‐type function, which was used to extrapolate the recrystallization behavior at room temperatures (dashed lines at the right top corner).

Figure 4a illustrates the spatial distribution of defects immediately following implantation (determined as being non‐diamond‐like) relative to the Sb atom position which is used to set the origin (0,0). Each of the two Sb atoms are included in the statistics as individual Sb atoms justified by the difference in the Sb_2_ binding energy and the implantation energy discussed above. The distribution is shown using cylindrical coordinates with the vertical *Z*‐axis passing through the dopant and extending perpendicularly to the surface. Each pixel is colored according to the density of the defects formed on average in a single cascade within a ring volume of thickness Δ*R* and height Δ*Z*, which are the increments of the *R*‐ and *Z*‐axis. *R* is the radius of the ring with respect to the position of the Sb atom. The defect density is shown according to the color bar from light (point defects) to dark (clusters) blue. The upper limit of the color bar was set to 15 defects nm^−3^, above which the defect clusters transform into amorphous pockets [34]. We note that in most of the simulations, Sb atoms are not located within the amorphous pockets but at the boundary of these. This observation aligns with an expected trend as heavy ions such as Sb tend to generate the most damage towards the end of their range, where many‐body collisions dominate. However, the Sb atoms themselves are not ‘caught’ within the most intense damage formation regions, and we also note that there is minimal damage below the location of the Sb atom.

Analysis of the damage cascade enables the size (number of atoms) of defect clusters formed to be studied. Figure 4b provides a histogram of defect cluster size distribution (immediately following implantation) showing that during ballistic phase a molecular ion produces about 10 point defects and less than 25 small defect clusters, while the majority of the defects (up to 94%) are bound in large amorphous pockets. In the inset of Figure 4b, one can also see that amorphous pockets with less than 500 defects form the most frequently. While large pockets with almost 10 000 defects may also form, they do so with much lower probability (a single case for 100 simulations). It has been shown previously that the activation energy for recrystallization of defective clusters in Si decreases with reducing cluster size, eventually decomposing into point defects that survive until they either vanish at the surface or recombine [34]. Hence here, we focus on the stability of the large amorphous pockets which we find typically occur fewer than ten times per implantation event. The size of these range from 30 to several thousand atoms and it is possible that these could form long‐lived stable amorphous regions.

To understand the long‐term stability of the ion induced defects further simulations were undertaken. As the MD simulations are limited to timescales of a few nanoseconds, we deduce the Arrhenius‐type temperature dependence of the time needed for recrystallization of individual amorphous pockets to 90% of their initial size. Simulations were performed at 700, 800, 900 and 1000 K with the decrease in cluster size with time fitted using a linear‐exponential function (Figure S1c). Figure 4c shows the temperature dependence of the recrystallization evolution for randomly selected large amorphous pockets across the range of simulated temperatures, Also shown in Figure 4c is the extrapolation of this temperature dependence down to 300 K. It is clearly seen that these pockets shrink to one‐tenth of their original size within seconds, even if simply left at room temperature.

The finding that the crystal lattice around a Sb dopant can recover fully within seconds without thermal annealing is aligned with the crystallinity seen in the ADF STEM images in Figure 3a,b. It is also known that the large fraction of crystal structure around an amorphous pocket enhances recrystallization further [35]. During a single ion impact, the damage is strongly localized and surrounded by a pristine crystal structure. This effect can be seen also in the difference of activation energies for recrystallization of the amorphous pockets located near the open surface and in the bulk, which we found in our MD simulations. Our results align with previous studies that reported lower activation energies for recrystallisation in smaller amorphous pockets compared to planar interfaces [33, 36, 37]. Near the surface, the effect of crystal structure around an amorphous pocket is weaker, and recrystallization is slower. A consequence of this recrystallization around the Sb is likely to lead its substitutional incorporation within the crystal structure, which is observed in the experimental images (e.g., Figure 3b). Nonetheless, in any application of this method, thermal annealing of the implanted Si would be undertaken thereby promoting electrical activation of dopants as is commonly performed, for example as in Jakob et al. [38].

To be utilized as solid‐state qubits, the isotopically selected Sb_2_ molecular ion must be implanted into Si deterministically with high confidence (detection efficiency, *η*). We have previously reported values of *η* = 87 (±7)% for 50 keV Sb ion implantation into using the P‐NAME tool [24]. However, *η* can vary significantly for different ion beam species, implantation energies, and target material combinations [39]. For the Sb in Si system considered here, measurements of *η* were undertaken using the method outlined by Cassidy et al. [39], more details of which are provided in Methods and by Adshead et al. [40].

**FIGURE 5:**
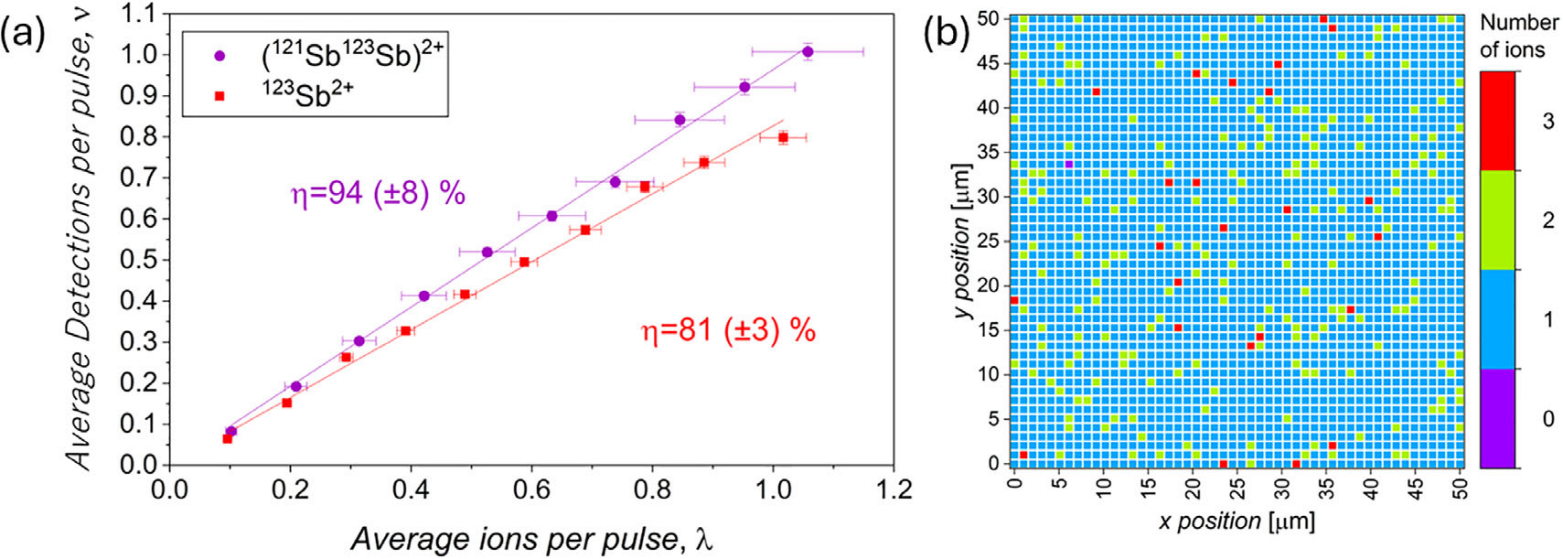
(a) Deterministic detection of efficiency of ^123^Sb^2+^ (50 keV) and (^121^Sb^123^Sb)^2+^ (50 keV) ions. (b) 50 × 50 array off ^123^Sb^2+^ ions obtained using λ = 0.1 ions pulse^−1^ showing the number of detected implantations at each point.

The implantation energies chosen for this investigation used the maximum anode voltage of the P‐NAME system (25 kV), which results in a nominal ion beam energy of 25 keV for a singly charged ion. However, as discussed above, there is no way to separate the isobars of the singly charged individual Sb ions from the doubly‐charged molecular ions (^121^Sb_2_^2+^ and ^123^Sb_2_^2+^) using the WF of the P‐NAME system. We therefore restrict the measurement of *η* to doubly charged ion species (^123^Sb^2+^ and (^121^Sb^123^Sb)^2+^) which can be isolated using the WF. Figure 5a shows the measurements from which η was determined for each of the implanted species. The implantation of the mixed‐isotope (^121^Sb^123^Sb) ^2+^ molecular ion exhibits the highest detection efficiency of *η* = 94 (±8)% compared to *η* = 70 (±5)% for a single ^123^Sb ion at the same implantation energy per atom (Table S1). When the implantation energy of the ^123^Sb^2+^ ion is increased to 50 keV (25 kV anode), the detection efficiency increases to *η* = 81 (±3)%, remaining significantly below that of the (^121^Sb^123^Sb) ^2+^ molecular ion (Figure 5a).

The correlative relationship between ion kinetic energy and secondary electron emission for ion‐substrate combinations has been well documented for a variety of ion‐material systems [41, 42, 43]. As such, an increase in detection efficiency by increasing the anode voltage from 12.5 to 25 kV for the doubly‐charged ion (increasing implantation energy from 25 to 50 keV) is to be expected. The increase of the secondary electron emission (indicated by an increased detection efficiency) for the mixed‐molecular ion species is also in line with previous work [44, 45], particularly with Sb molecular ions [46]. In this work Veje et al. found that an increase in Sb. molecular ion size leads to an increase in secondary electron emission, though with a sublinear correlation between molecular ion size and average secondary electron yield per atom within the molecular ion.

Finally, we apply the utilization of deterministic doping of ^123^Sb ^2+^ ions to form extended arrays. In this case, ^123^Sb ^2+^ was investigated as opposed to (^121^Sb^123^Sb) ^2+^ in order to study the effect of single ion implantation and the distinct signals generated from these ions rather than the signals from molecular ions, which are expected to have more complex correlation in nature between peak numbers and number of molecules. Using pulsed ion control with *λ* ≈ 0.1 ions pulse^−1^ a 50 × 50 doped array of single ions was attempted. Figure 5b shows the recorded detections at each site, and Table S2, the total number of pulses used to complete the array, and the number of peaks detected for each of the successfully detected pulses. Deterministic detection clearly enables arrays free from empty points to be created (the dark count rate which may lead to a false positive detection is ≈8 × 10^−5^ counts per second for the P‐NAME system) [24]. By reducing λ, it is possible to reduce the probability of sites where a pulse of more than one ion is implanted. This logic is accurate for both single atomic ion implantations, as well as molecular ion implantation, as the detection efficiency refers to the likelihood of being able to detect a single “implantation event”. The method used to create the array is fully scalable without restriction to produce arrays in excess of 1 million dopants.

## 3 Conclusion

This study has demonstrated the ability to isolate isotopically unique (^121^Sb^123^Sb) molecular ions using a liquid metal alloy ion source. NanoSIMS analysis demonstrates isotopic doping of ^121^Sb^123^Sb atoms into Si with the expected ^121^Sb:^123^Sb ratio using the P‐NAME system. Atomically resolved imaging of implanted pairs of ^121^Sb^123^Sb atoms reveals the expected atomic distribution and separations consistent with theoretical calculations. Molecular dynamic simulations reveal that the ion damage caused by the cluster implantation is rapidly recrystallized within seconds, even without a thermal anneal above 300 K. This process likely leads to the Sb atom being substitutionally incorporated within the Si lattice as observed in ADF STEM imaging. The observed isotopically selected ^121^Sb^123^Sb atomic pairs with mean separations of ≈2 nm following implantation are ideally suited to form coupled qudit systems due to overlap of their respective electronic wavefunctions. The effective demonstration of this for Sb ions provides the possibility to explore higher dimensional Hilbert spaces in the 3‐in‐1 qubit regime, previously only demonstrated with P. Analysis of the detection efficiency of single (^121^Sb^123^Sb)^2+^ molecular ion implantation with P‐NAME yields a value of η = 94 (±8)%, showing that deterministic doping of single molecular ions with high confidence is feasible. Coupled with the ability of the same instrument to also provide ≈2 ppm ^29^Si host regions for these to reside [18], we here demonstrate a scalable pathway for fabricating large arrays of qudits. These results open up a new route to optimized Si‐based quantum computing platforms.

## 4 Methods

Implantation was performed using a mass‐selected Sb ion beam provided by the Platform for Nanoscale Advanced Materials Engineering (P‐NAME, Q‐One Ionoptika) tool whose details are reported in full elsewhere [24]. The in‐column Wien filter was used to mass‐select Sb ions with an optimum mass resolving power (*R* = *M*/Δ*M*) of 296 ± 7 for the Sb^+^ isotopes under mid‐current (in the range of 10's pA) mode at a 25 kV anode voltage. Implanted substrates were placed with a 3° offset with respect to the beam in order to minimize ion channeling.

The source alloy had Au_73_Si_14_Sb_13_ near‐eutectic composition produced by arc melting 99.9% gold ingot (Cookson Precious Metals Limited) with 99.99% Si and 99.5% Sb lump (both Thermo Fisher Scientific). Arc melting was conducted in 30 mTorr vacuum, backfilled with Ar on a Cu hearth. The liquid alloy was flipped three times, and then the power was stopped and the alloy solidified on the water‐cooled Cu plate.

NanoSIMS analysis was performed using a NanoSIMS 50L (CAMECA, France), employing a 16 keV Cs^+^ primary ion beam with a beam current of 0.4–0.5 pA and approximate beam size of 75–100 nm. It was scanned across the sample surface to generate negative secondary ions, with secondary electron (SE) images also collected. The instrument was tuned to achieve a R exceeding 6000 for all detectors, effectively mitigating any mass interferences. A double‐focusing mass spectrometer facilitated the simultaneous detection of key ions ^12^C, ^16^O, ^28^Si, ^31^P, ^121^Sb, ^123^Sb and SE. Imaging was conducted over 500 planes, each with a 256 × 256 pixel resolution and a 5 µm raster size, with a dwell time of 1000 µs pixel^−1^. To enhance spatial resolution, the D1 aperture is adjusted to D1‐4 (150 µm diameter) and entrance slits to ES‐3 (30 µm width), alongside an aperture slit set to AS‐2 (200 µm width). The NanoSIMS signals in Figure 2 have been normalized to the native ^28^Si signal taken from a depth where there is no further Sb signal observed. It is observed that the ^123^Sb signal is reproducibly higher across all samples, by about 13% which is accounted for by a systematic instrumental effect relating to the exact location of the collected mass. This was intentionally introduced to reduce mass interference in the NanoSIMS measurements and does not affect the results obtained. Atomic force microscopy (AFM) profiling was conducted using a nanoSurf CoreAFM with a tapAL‐190 probe and was used the calibrating the NanoSIMS etch profiles.

Implantation for STEM was carried out into 20 nm thick Si membranes (Silson) using a 15 kV anode voltage, 30 keV implantation energy, Sb molecular ion beam. STEM characterization used a Thermo Fisher Titan equipped with a Schottky field emission gun (FEG). STEM images were acquired using a ThermoFisher Titan ChemiSTEM at an accelerating voltage of 200 kV with a probe semi‐convergence angle of 21 mrad, a probe current of 78 pA. Annular dark field (ADF) imaging was performed with detector inner/outer angles of 54/200 mrad and an electron fluence of 1950 e^−^Å^2^. Further STEM images, were acquired using a ThermoFisher Iliad STEM with an accelerating voltage of 300 kV, a probe semi‐convergence angle of 21.4 mrad, a probe current of 14 pA. ADF imaging used inner/outer collection angles of 33–200 mrad and an electron fluence of 783 e^−^Å^2^ per image, requiring a relatively high signal‐to‐noise (S/N) to confidently resolve the implanted species with respect to the silicon. Nonetheless, it is desirable to limit the electron fluence since the high energy electron beam has the potential to cause displacement of the single‐ion dopants. Immediately prior to STEM imaging the implanted membrane samples were cleaned using toluene at 50°C to remove any volatile organic compounds adsorbed on the surface. Multislice simulations were performed with abTEM [47] using the Kirkland parametrization [48, 49, 50] and PRISM algorithm [51]. For further information, see Section S4.

TRIDYN simulations were performed using the tridyn2020l version of the code. The input files were modified as described in the text, ensuring that each simulation achieved a maximum relative areal density change per pseudoprojectile of <1%, as required to ensure statistical validity of the simulation.

Theoretical modelling of radiation damage creation and annealing at room temperature was performed using the classical molecular dynamics package LAMMPS [52], allowing bond breaking, recoils, and defect formation to occur naturally. Si‐Si interactions were described using the Stillinger‐Weber potential [53] while Sb–Sb and Sb–Si interactions were modelled by a Lennard‐Jones potential [54] and a purely repulsive ZBL potential [55] respectively. Electronic stopping was modelled by a friction force proportional to the velocity [55, 56]. MD simulations were conducted using a 21.7 × 21.7 × 65.7 nm^3^ cell to capture ion channeling effects. Even with such a large cell, occasional channeling cases were recorded reaching the boundary. The simulations applied NVT thermostat cooling at 300 K (τ = 0.1 ps) to a 0.2 nm boundary layer on the sides and bottom of the cell, with an additional 2 Å frozen layer beneath to prevent drift. The interior atoms evolved under NVE conditions. To avoid reinsertion due to periodic boundaries, a damping force was applied when energetic atoms approached the XY borders. Each simulation ran for 15 ps, by which time the generated damage was considered stable on the MD timescale.

Postprocessing was performed using OVITO [57]. Defects were labelled as “OTHER” type in Diamond Structure analysis and were grouped into damage clusters with an 8.1 Å cutoff. To obtain the recovery time of the crystal lattice, selected pockets were extracted into smaller cells. The cut was performed so that it keeps the crystalline diamond structure around the cluster and at the periodic boundaries. These cells were simulated at different temperatures generating initial velocity distributions randomly and repeating five times per temperature and cluster, to obtain statistics. The number of defects in the cell was monitored, and the simulation was stopped when the original number of defects had decreased nine tenths of the original number.

The detection efficiency measurements were conducted using the P‐NAME internal high sensitivity secondary electron detectors. The ion beam currents were 105 (±4) fA, 86 (±6) fA, and 86 (±7) fA for the 50 keV monoatomic ion, 25 keV monoatomic ion, and 50 keV mixed molecular ion, respectively. The electrostatic pulser voltage (nominally high to keep the beam blank) was dropped to 0 V for a desired set time (the pulse width) such that the average number of ions per pulse is Poissonian in nature. The pulse width was varied for the average number of ions per pulse from 0.1 to 1, in 0.1 steps. When the beam was pulsed, the ion implanted into the substrate releases secondary electrons into the chamber, which are collected by the secondary electron detectors. The efficiency of this collection is dominated by the secondary electron emission, which is ion beam and material dependent.

In order to calculate the efficiency, the number of pulses required to achieve a total of 2500 successful implantation detections (secondary electron signals correlating with the opening of the electrostatic pulser) was recorded, and linear regression applied to the data [39] to determine the detection efficiencies for the different ion species. The ions were implanted in a 50 × 50 array with a pitch of 1 um between each implant, and each of the variations in pulser duration and ion beam species was implanted into a new region of the Si to avoid any potential effect of localized charging or secondary electron depletion. The dak count rate (false positive detections) of the P‐NAME system was measured to be *k* = 8 × 10^−5^ s^−1^, and so not considered to have a significant impact on the measurements made in this work.

## Author Contributions

R.J.C. and S.J.H. conceived and supervised the project. M.A. and M.C. performed ion beam doping, simulation and contributed to SIMS analysis. K.M. supervised NanoSIMS analysis. K.L. performed NanoSIMS analysis. E.T. and R.E. performed TEM imaging and analysis. W.T. and S.S‐A. contributed to TEM analysis. C.M.G. supervised and contributed to alloy development. Y.C. contributed to alloy development. F.D. supervised and contributed to the molecular dynamic simulations. T.B. and A.M. contributed to the molecular dynamic simulations. All authors contributed to manuscript writing and editing.

## Funding

The authors acknowledge funding from the Engineering and Physical Sciences Research Council (EPSRC) for funding under grants EP/Y024303, EP/S021531/1, EP/M010619/1, EP/V007033/1, EP/S030719/1, EP/V001914/1, EP/V036343/1 and EP/P009050/1, and also for the EPSRC Centre for Doctoral Training (CDT) Graphene‐NOWNANO (EP/L01548X/1). TEM and NanoSIMS access was supported by the Henry Royce Institute for Advanced Materials, funded through EPSRC grants EP/R00661X/1, EP/S019367/1, EP/P025021/1 and EP/P025498/1. The NanoSIMS was funded by UK Research Partnership Investment Funding (UKRPIF) Manchester RPIF Round 2. S.J.H. and E.T. acknowledge funding from the European Research Council (ERC) under the European Union's Horizon 2020 research and innovation programme (Grant ERC‐2016‐STG‐EvoluTEM‐715502).

## Conflicts of Interest

R.E. was an employee of Thermo Fisher Scientific at the time of this work being conducted, and a Thermo Fisher Scientific instruments were used to acquire the STEM images reported.

## Supporting information




**Supporting File 1**: adma72697‐sup‐0001‐SuppMat.docx.

## Data Availability

The data that support the findings of this study are available from the corresponding author upon reasonable request.
