# Supplementary material for: Isotopically Selected Co‐Doping of 121Sb and 123Sb Pairs in Silicon
Source: Adv Mater. 2026 Mar 2;38(18):e22240. doi: 10.1002/adma.202522240 (PMC13014029; doi:10.1002/adma.202522240)
Supplement: Supplementary file 1 — Supporting File 1: adma72697‐sup‐0001‐SuppMat.docx. [file ADMA-38-e22240-s001.docx]

Supporting Information

Isotopically Selected Co-Doping of ^121^Sb and ^123^Sb Pairs in Silicon

Mason Adshead, Maddison Coke, Evan Tillotson, Tomas F Bouvier, Artem Mkrtychyan, Kexue Li, Sam Sullivan-Allsop, Ricardo Egoavil, William Thornley, Yi Cui, Christopher M. Gourlay, Katie L Moore, Flyura Djurabekova, Sarah J Haigh, Richard J Curry*

**SI Section 1) Molecular Dynamics Simulation**

Figure S1. a) Snapshots at diﬀerent times of the damage cascade induced by a (^121^Sb^123^Sb)^2+^ molecular ion implantation at 30 keV in Si. White atoms represent "non-diamond atoms" identified via Diamond Structure analysis in OVITO. The blue and red large-size atoms show the final position of two Sb atoms. A temperature heat map is superimposed on the particle slice. b) Temporal evolution of the number of defects during the implantation as the percentage with respect to the stable number of defects at the end of the simulation with a 1σ confidence band. c) Defect evolution during annealing simulations of the cluster, cropped from the final cascade configuration a. A combined linear-exponential model was fitted to the data, with a 1σ band included.

**SI Section 2) Wein Filter Scans of AuSiSb source**

**
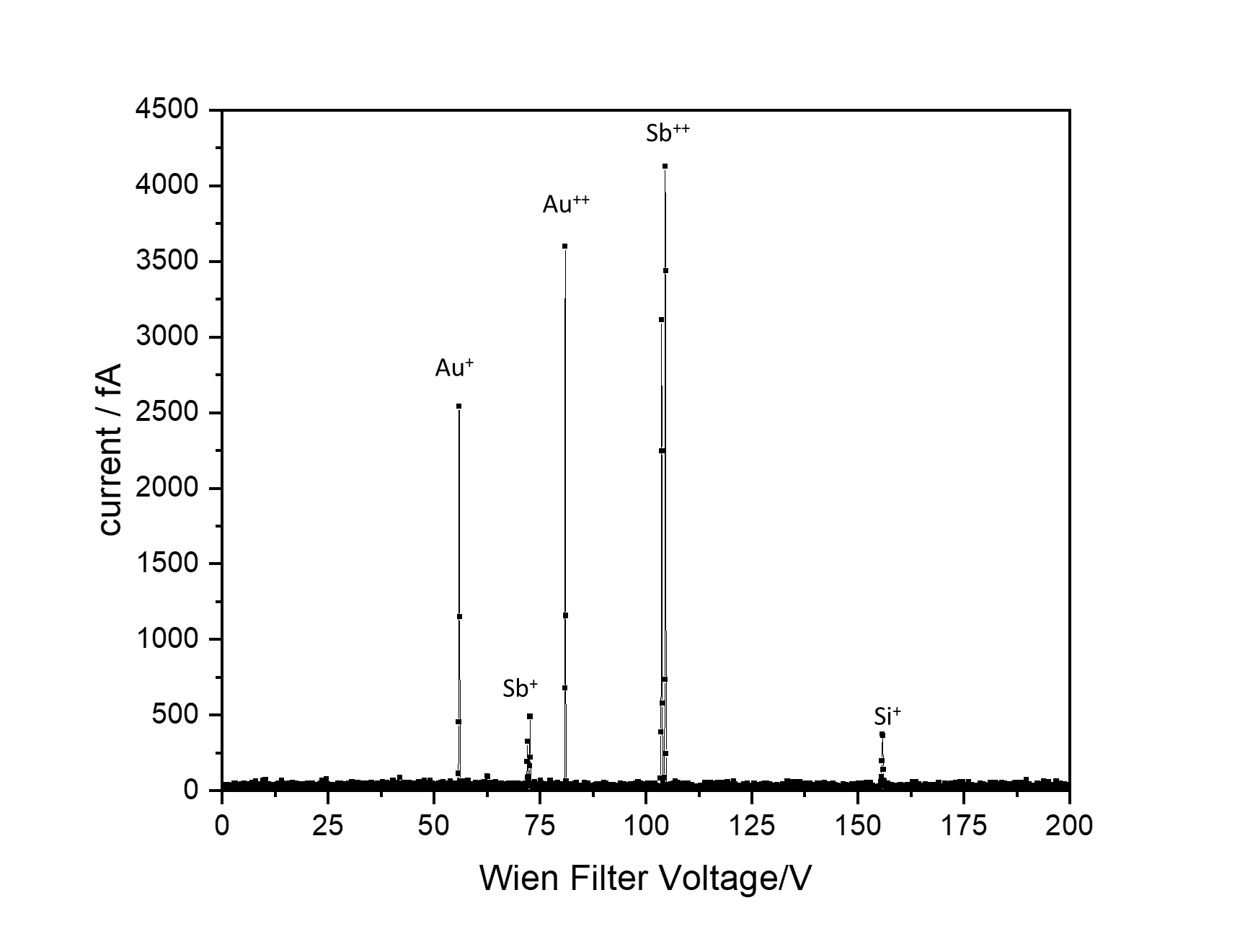
**

Figure S2. Wien filter (WF) scan of the AuSiSb liquid-metal-alloy source emission at 15 kV anode voltage, high mass resolution, low dose mode. The measured data (black squares) are fitted with line as guide to the eye. Those species labelled (Sb^+^, Au^++^ and Sb^++^) have been verified experimentally, clusters with higher mass only visible in high dose mode and therefore not shown here.

**SI Section 3) Electron energy loss spectroscopy (EELS) characterization of Sb-implanted 20-nm Si membranes**

**
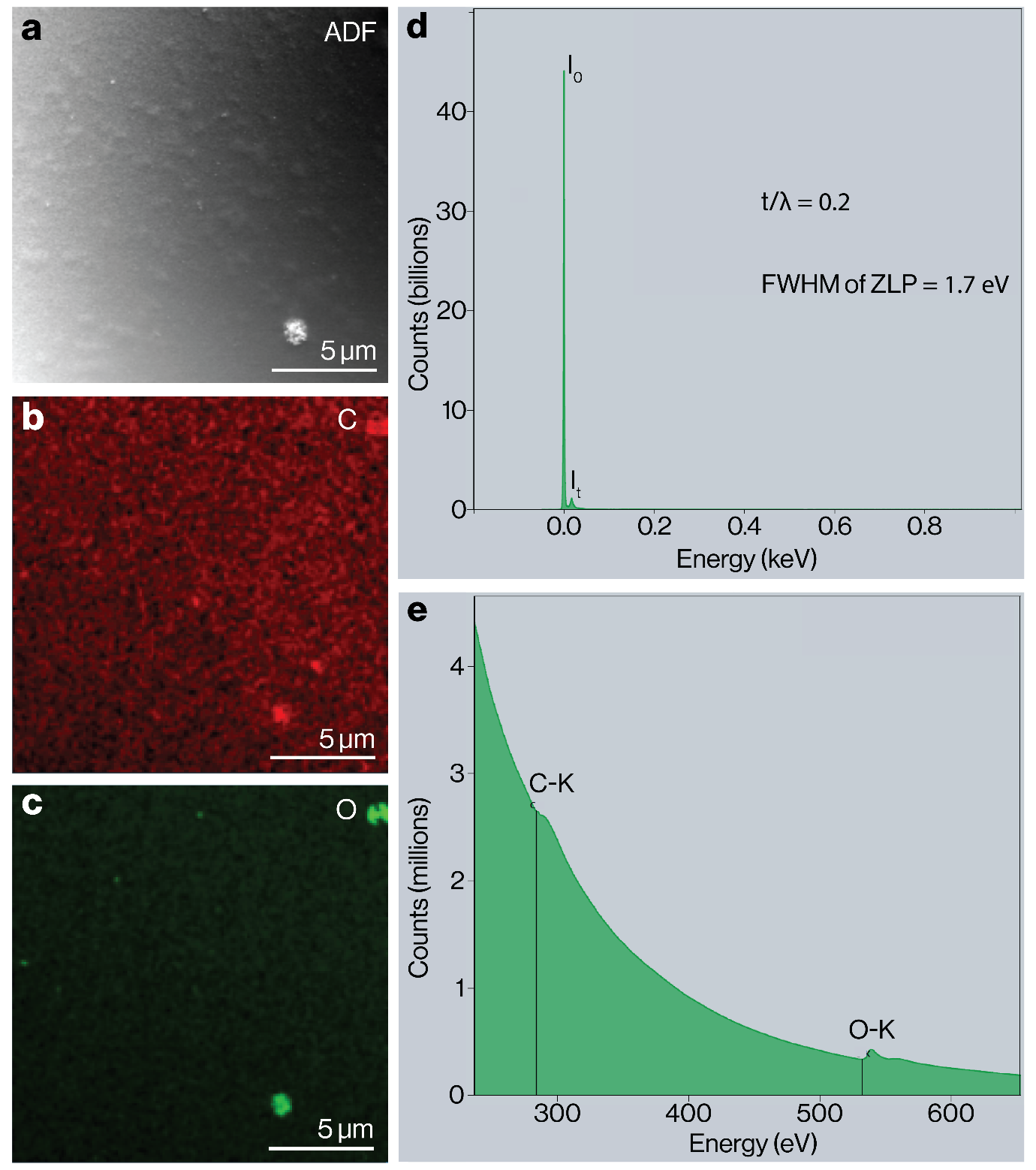
**

Figure S3. Electron energy loss spectroscopy (EELS) characterization of 20-nm Si membrane. a)-c) Annular dark field (ADF) image with accompanying carbon and oxygen elemental maps, respectively. d) Full EELS spectra with log-EELS technique showing relative thickness of 0.2 for the Si membrane, calculating a real thickness of 22.6 nm for 300 kV mapping [1], [2]. e) Magnified EELS spectra showing carbon and oxygen peaks.

**SI Section 4) Multislice simulations of Sb-implanted 20-nm Si membranes**

The following multislice simulations have been performed with abTEM [3] using the Kirkland parametrization [4]–[6] and PRISM algorithm [7]. The acceleration voltage was modelled at 200 kV with 143 slices of 1.4 Å thickness, comprising the 20-nm implanted Si membrane. Simulations were performed with the Sb-ions implanted at the bottom, middle and top sections of the Si membrane, with the probe focused at the top of the specimen. Three separate convergence angles of probe (21.4, 25 and 30 mrad) have been modelled for three diﬀerent collection angle ranges, 0-convergence, 30-50 and 54-200 mrads, corresponding with bright field (BF), low-angle annular dark field (LAADF) and high-angle annular dark filed (HAADF) electron signals, respectively. The collated data is shown below in Figure S3, with the full simulations for the three convergence angles shown in Figure S4 and Figure S5.


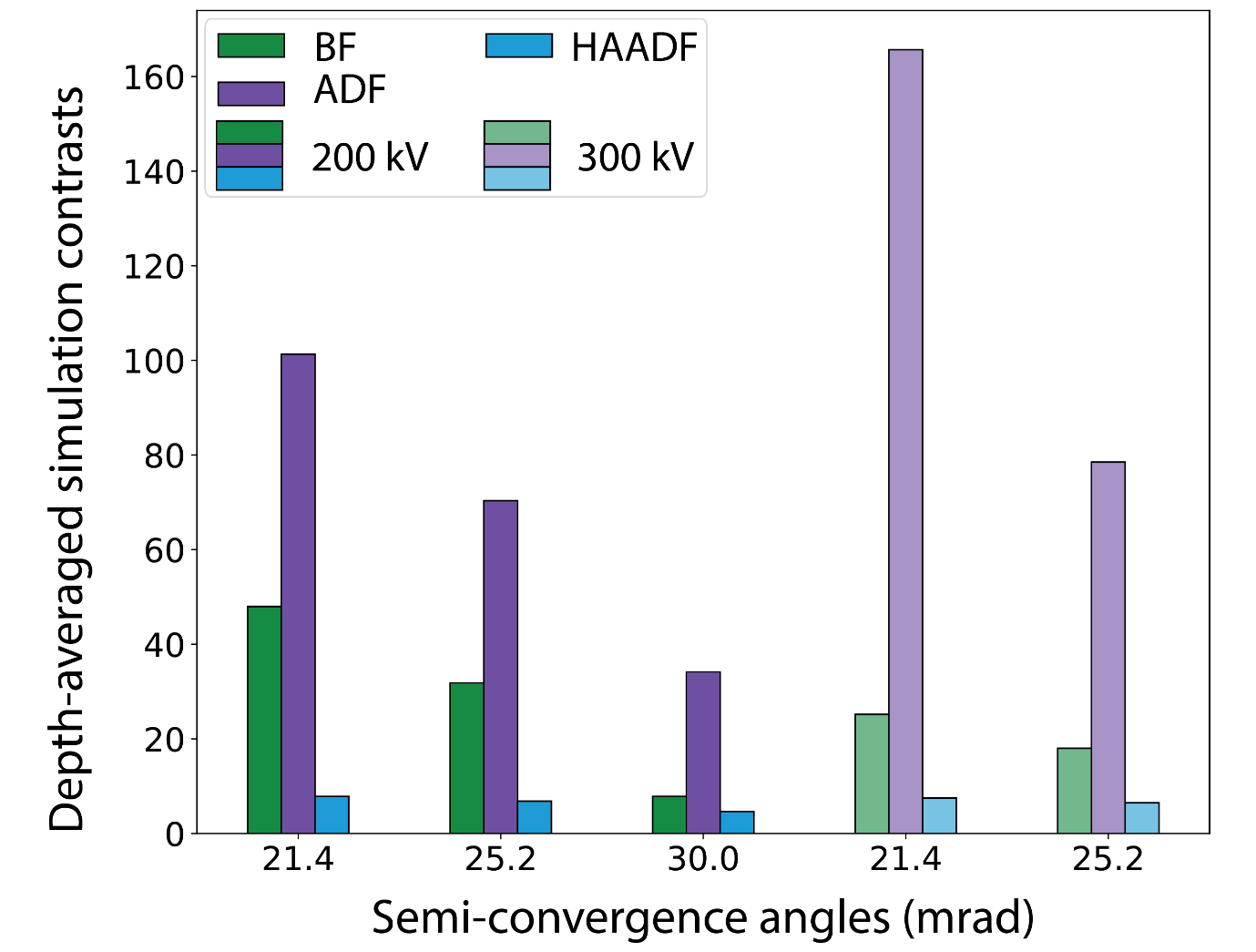


Figure S4. Contrasts of dopant atom against lattice atoms as a function of collection and convergence angles for 20nm-thick Sb-implanted Si membranes. The contrasts are averaged over depth of the implanted dopant at three positions (top, middle and bottom). Annular dark field (ADF) imaging at 300 kV predicts the greatest average contrast of dopant.

**
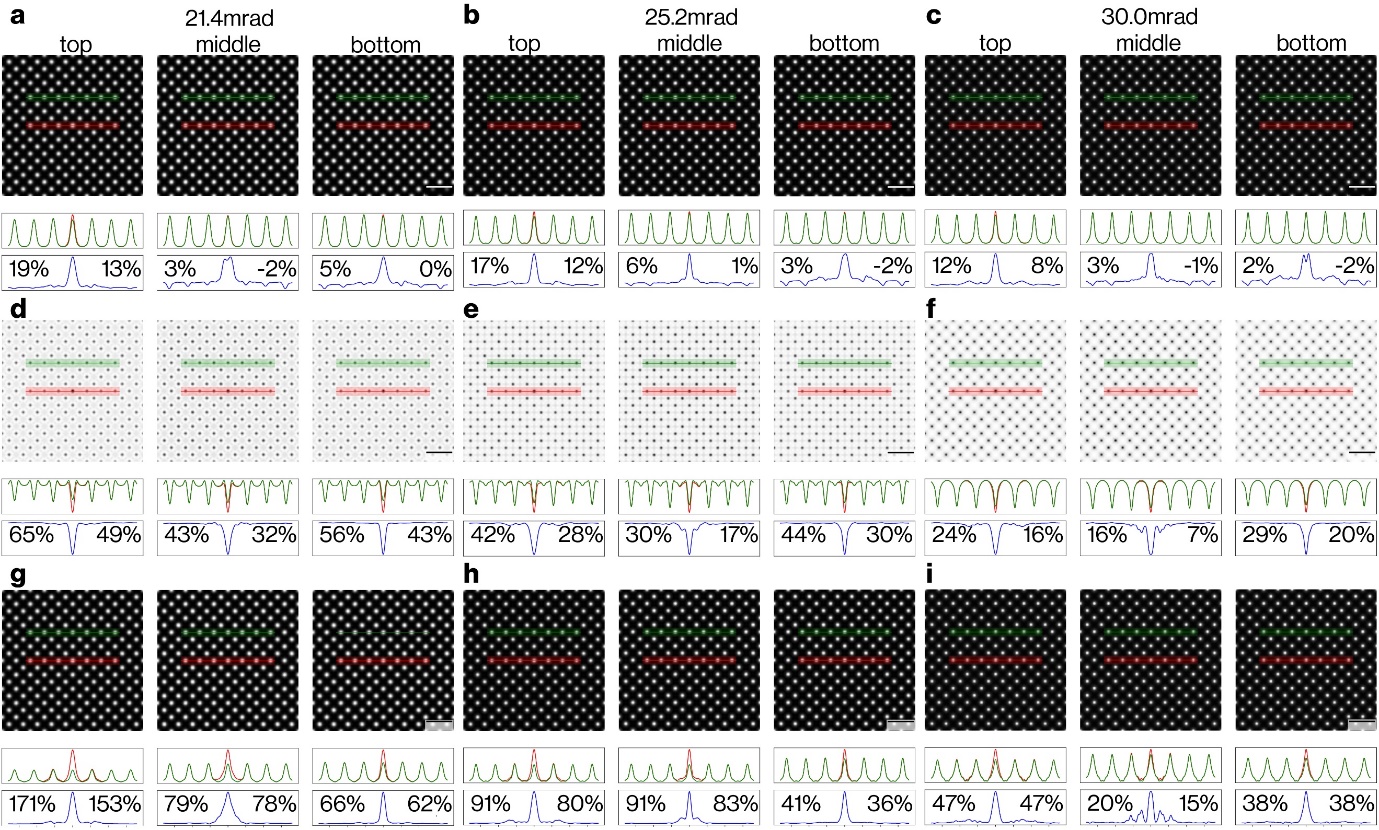
**

Figure S5. Full 200 kV Multislice simulations. a)-c), High-angle annular dark field (HAADF), d)-f) bright field (BF) and g)-i), annular dark field (ADF) images for semi-convergence angles of 21.4, 25.2 and 30 mrad, respectively. The scale bars are 500 pm.

**
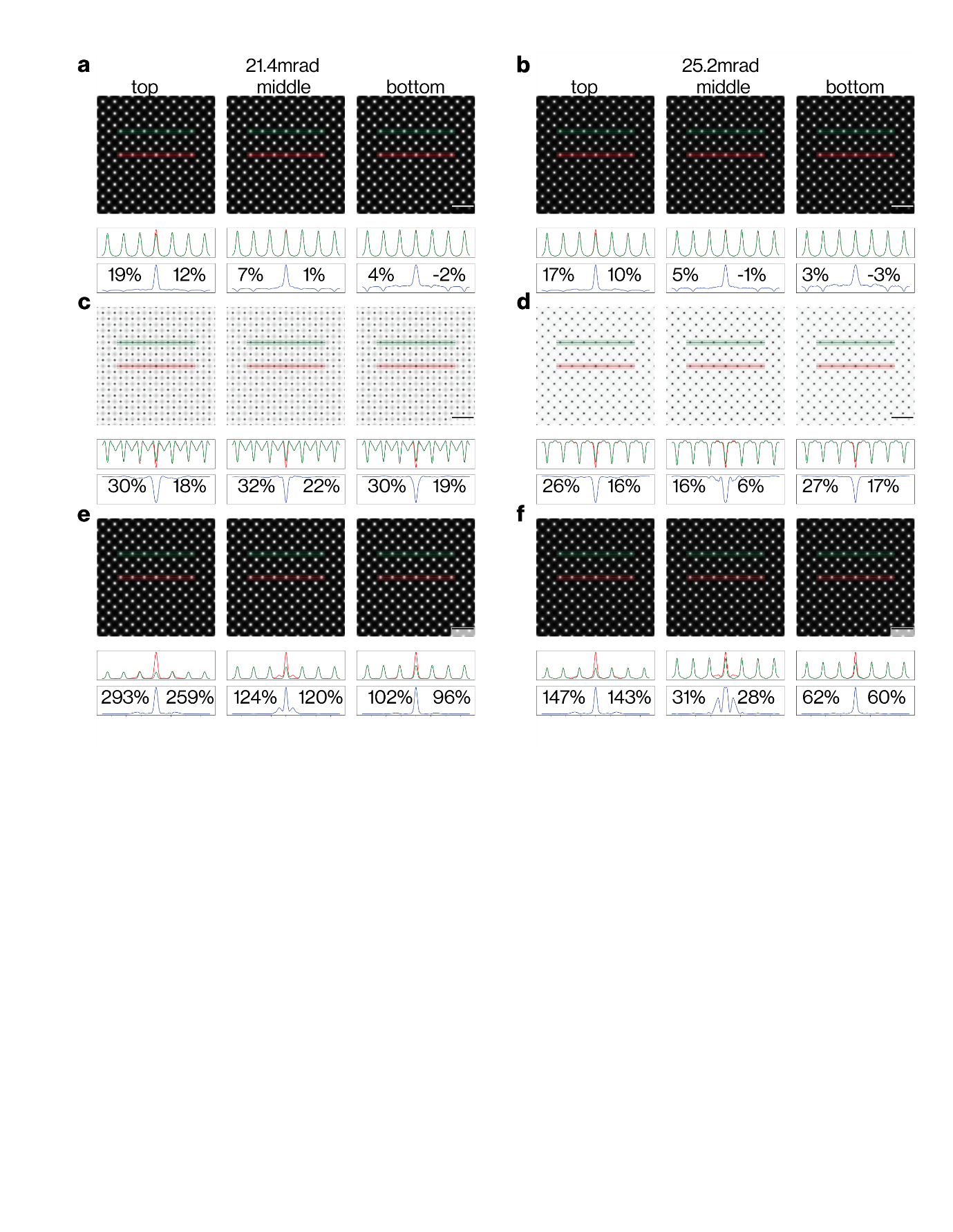
**

Figure S6. Full 300 kV Multislice simulations. a)-b), High-angle annular dark field (HAADF), c)-d) bright field (BF) and e)-f), annular dark field (ADF) images for semi-convergence angles of 21.4 and 25.2 mrad, respectively. The scale bars are 500 pm.

**SI Section 5) Single ion implantation detection eﬃciencies**

Table S1. Detection eﬃciency measurements of three antimony ion species.

| Species | Anode voltage (kV) | Charge state | Energy per ion (keV) | η (%) |
| --- | --- | --- | --- | --- |
| ^123^Sb^121^sb | 25 | 2+ | 25 | 94 (± 8) |
| ^123^Sb | 25 | 2+ | 50 | 81 (±3) |
| ^123^Sb | 12.5 | 2+ | 25 | 70 (±5) |

Table S2. Statistical data for 50 x 50 ^123^Sb^2+^ array. λ has been adjusted to take the switching latency into account [8].

| λ (ions/pulse) | Number of peaks detected | | | | | | | |
| --- | --- | --- | --- | --- | --- | --- | --- | --- |
|  | **0** | **1** | **2** | **3** | **4** | **5** | **6** | **7** |
| 0.096 | 37523 | 2286 | 189 | 24 | 0 | 0 | 0 | 0 |
| 0.194 | 15265 | 2191 | 271 | 37 | 1 | 0 | 0 | 0 |
| 0.292 | 8310 | 2109 | 334 | 53 | 3 | 1 | 0 | 0 |
| 0.391 | 6457 | 2004 | 424 | 60 | 10 | 1 | 0 | 0 |
| 0.489 | 4837 | 1939 | 468 | 83 | 8 | 2 | 0 | 0 |
| 0.587 | 3901 | 1799 | 561 | 115 | 20 | 4 | 0 | 0 |
| 0.689 | 3229 | 1764 | 564 | 132 | 31 | 7 | 1 | 1 |
| 0.787 | 2577 | 1661 | 617 | 182 | 32 | 6 | 1 | 1 |
| 0.886 | 2290 | 1582 | 651 | 204 | 48 | 12 | 2 | 1 |
| 1.017 | 2047 | 1520 | 677 | 235 | 54 | 12 | 1 | 0 |


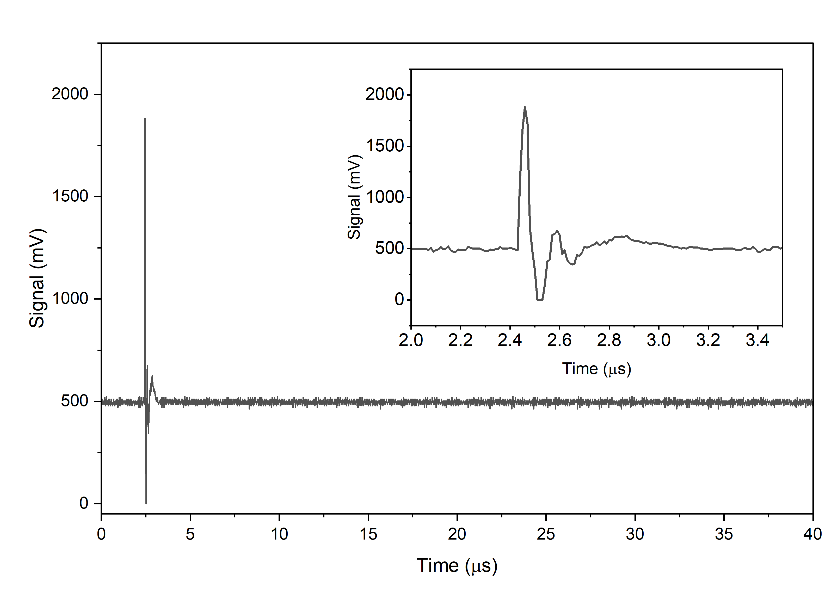


Figure S7. Example single ion detection peak with black level (baseline) adjusted to show the characteristic voltage dip and recovery for a positive detection.

**References**

[1] T. Malis, S. C. Cheng, and R. F. Egerton, “EELS log‐ratio technique for specimen‐thickness measurement in the TEM,” *J. Electron Microsc. Tech.*, vol. 8, no. 2, pp. 193–200, 1988.

[2] H. Meltzman et al., “An experimental method for calibration of the plasmon mean free path,” *J. Microsc*., vol. 236, no. 3, pp. 165–173, 2009.

[3] J. Madsen and T. Susi, “The abTEM code: transmission electron microscopy from first principles,” *Open Res. Eur*., vol. 1, p. 24, 2021.

[4] E. J. Kirkland, “Computation in electron microscopy,” *Acta Crystallogr. Sect. A Found. Adv*., vol. 72, pp. 1–27, 2016.

[5] E. J. Kirkland, R. F. Loane, and J. Silcox, “Simulation of annular dark field stem images using a modified multislice method,” *Ultramicroscopy*, vol. 23, no. 1, pp. 77–96, 1987.

[6] E. J. Kirkland, Advanced Computing in Electron Microscopy, no. Second Edition. Boston, MA: Springer US, ISBN: 978-1-4419-6532-5, 2010.

[7] C. Ophus, “A fast image simulation algorithm for scanning transmission electron microscopy,” *Adv. Struct. Chem. Imaging*, vol. 3, no. 1, pp. 1–11, 2017.

[8] M. Adshead, L. K. Wan, M. Coke, R. J. Curry "Deterministic detection of single ion implantation", arXiv:2510.01035 [cond-mat.mtrl-sci].
